# Community Readiness for Increasing Older Adult Physical Activity Levels in Kazakhstan

**DOI:** 10.5195/cajgh.2020.447

**Published:** 2020-03-31

**Authors:** Aniyar Izguttinov, Assel Ainabekova, Miruna Petrescu-Prahova, Suzanne J. Wood

**Affiliations:** 1 Department of Health Policy and Management, University of North Carolina at Chapel Hill, Chapel Hill, North Carolina, USA; 2 Center for Global Health, Republican Center for Health Development, Ministry of Healthcare of the Republic of Kazakhstan, Nur-Sultan, Kazakhstan; 3 Department of Health Services, University of Washington, Seattle, Washington, USA

**Keywords:** Community readiness, Physical activity, Older adults, Healthy aging

## Abstract

**Introduction:**

Physical activity is proven to be a significant element of successful aging, but many seniors worldwide fail to achieve the recommended levels. This study aimed to assess the readiness of the community in Nur-Sultan, Kazakhstan, to act on the issue of physical inactivity among older adults.

**Methods:**

In order to achieve this purpose, we conducted qualitative interviews with key informants in the community and applied a validated community readiness tool.

**Results:**

The results suggest that the local community is at early stages of readiness to act on the issue of older adult physical inactivity. We identified a number of barriers that prevented seniors from leading active lifestyles, which included community misconceptions about older adult physical activity, family centeredness in older adulthood, scarcity of resources, passive support from the leadership, and lack of efforts in the community. Research findings also highlighted the importance of conducting in-depth analysis of key informant responses in addition to calculating readiness scores, when using the community readiness tool.

**Conclusions:**

Community-specific strategies for enhancing the level of physical activity among seniors are required to offset the disease burden associated with aging and to prolong life expectancy in Kazakhstan, and it is of paramount importance to tailor potential efforts as to address the current readiness of the community and its needs.

Physical activity is proven to be a significant element of successful aging.^1^ Apparent benefits of physical activity for older adults include an increase in functional ability and reduction in the risk of cognitive decline.^2^ Scientific evidence also suggests that physical activity prevents onset of diabetes and stroke, improves sleep and life satisfaction, and helps to build social networks in older adulthood.^3^ Despite these benefits, many seniors fail to achieve the recommended levels of physical activity.^4^–^6^ One clear finding in the literature is that inactivity increases substantially with age across nations.^3^,^7^

The Kazakhstan of today has improved in many health status measures relative to the 1990s, when the country obtained its independence. In 2017, the average life expectancy was 72.9 years, which indicates a three-and-a-half-year gain compared to the 1980s.^8^ However, cardiovascular diseases in particular place the greatest burden on the population of Kazakhstan, accounting for 53% of mortality in the nation.^9^ An analysis across age groups demonstrates an even more alarming picture, with cardiovascular diseases estimated to be the single leading cause of deaths for the age groups of 45–59 and 60–74 years.^9^

Given the high prevalence of cardiovascular diseases, especially in older age, and relatively short life expectancy in Kazakhstan, increasing physical activity levels among older adults in the country appears to be a very promising public health measure.^3^ However, little is known about the readiness of communities in Kazakhstan to implement older adult physical activity initiatives, and the design of successful programs will have to consider current community norms and activities. Therefore, the purpose of this study was to systematically assess the community readiness to act on the issue of physical inactivity among older adults aged 60 and over in the city of Nur-Sultan (formerly Astana), Kazakhstan. We believe the study findings will help inform locally tailored initiatives to enhance older adult physical activity levels and will facilitate the adoption of evidence-based public health programs in the region.

## Methods

A cross-sectional community readiness assessment was carried out to achieve the purpose of the study. The community was defined by the geographical area of the city of Nur-Sultan.

### Community Readiness Tool

Community readiness (CR) is defined as the degree to which a certain community is willing and prepared to take action on a specific health problem.^10^ We chose the Community Readiness Tool (CRT) developed by Edwards et al.^11^ as the assessment tool for our study because it (1) offered flexibility in tailoring the approach to a particular health issue and a community; (2) has been used successfully to analyze potential dissemination of older adult physical activity programs in the US, Germany, and China;^12^–^14^ and (3) previous studies have reported the validity and high consistency of the tool.^15^

The CRT provides a step-by-step protocol for the assessment of five dimensions of CR: (1) Community Knowledge of Issue, (2) Community Knowledge of Efforts, (3) Community Climate, (4) Leadership, and (5) Resources. All dimensions are scored separately using a nine-point anchored rating scale before an overall numeric value is calculated. Each score on the scale corresponds to one of the nine stages of CR,^10^ which are described in Table 1.

### Participant Recruitment

The purposeful sampling was used to recruit key informants from a diverse range of community sectors.^16^ Through online search and personal connections, we identified and contacted seventeen potential interviewees. The CRT suggests interviewing 6-12 key informants depending on the size of the community. In this study, the final sample included ten (N=10) individuals representing five different sectors: (1) older adult organizations, (2) public health agencies, (3) fitness/sports facilities, (4) healthcare providers, and (5) social service organizations. The team did not offer any incentives for participation.

### Interview Guide and Procedures

The interview instrument was developed in accordance with the CRT and directly addressed all five dimensions. The final version of the guide was translated into the Kazakh and Russian languages and pilot-tested. All interviews were held in-person between June and December 2018 and followed a semi-structured format.^17^ The team obtained a written consent from each informant. While interviewees were given a choice of three languages (Russian, Kazakh, and English), all of them preferred to answer the questions in Russian. Each interview, which lasted 45–70 minutes, was audio recorded with permission, and then transcribed using the online transcription software HappyScribe®.

**Table 1.** Stages of community readiness

Stages	Title	Description
1	No awareness	Issue is not generally recognized by the community or leaders as a problem (or it may truly not be an issue).
2	Denial/resistance	At least some community members recognize that it is a concern, but there is little recognition that it might be occurring locally.
3	Vague awareness	Most feel that there is a local concern, but there is no immediate motivation to do anything about it.
4	Preplanning	There is clear recognition that something must be done, and there may even be a group addressing it. However, efforts are not focused or detailed.
5	Preparation	Active leaders begin planning in earnest. Community offers modest support of efforts.
6	Initiation	Enough information is available to justify efforts. Activities are underway.
7	Stabilization	Activities are supported by administrators or community decision makers. Staff are trained and experienced.
8	Confirmation/ expansion	Efforts are in place. Community members feel comfortable using services, and they support expansions. Local data are regularly obtained.
9	Community ownership/ Professionalization	Detailed and sophisticated knowledge exists about prevalence, causes, and consequences. Effective evaluation guides new directions. Model is applied to other issues.

### Analysis

Although ten interviews were conducted, we excluded one from the analysis due to lack of analyzable responses and failure to provide information for scoring two of the CRT dimensions. Hence, a total of nine (N=9) interview transcripts were subject to analysis.

We first assessed participant characteristics and CR scores closely following the CRT and using the anchored rating scales that are part of the tool.^10^ Two authors independently scored each interview before discussing individual assessment discrepancies and producing a final table with consensus scores. As recommended by Kostadinov et al.,^18^ we also calculated standard deviations of CR scores.

For the purposes of qualitative data analysis, we developed an a-priori list of codes to conduct a deductive assessment based on the CRT and the interview guide. Then, using an inductive approach,^19^ we coded two previously translated interview transcripts in order to identify any emergent codes. The final codebook, containing 29 a-priori and four emergent codes, was used to code the entire dataset. Qualitative coding results were then discussed with the whole research team and eventually the themes were mapped onto one of the five CR dimensions. Dedoose® Version 8.1 was used for qualitative data analysis.

## Results

### Participant Characteristics

All participants were directly involved in the provision of services to senior citizens or were actively engaged in policy issues that potentially affected older adult physical activity levels. Seven informants were female (78%) and two were male (22%). While the majority of interviewees were between 30 and 50 years old, two participants were over 60 years old. Table 2 describes the sample characteristics.

**Table 2.** Key informant characteristics (N=9)

Characteristic	n (%)
**Gender**	
Male	2 (22)
Female	7 (78)
**Age category**	
30–44 years	5 (56)
45–59 years	2 (22)
>60 years	2 (22)
**Representative from:**	
Older adult organization	1 (11)
Public health agency	2 (22)
Fitness / sports facility	3 (34)
Hospital / Healthcare provider	2 (22)
Social service organization	1 (11)
**Representative job title:**	
Director / Deputy director	2 (22)
Head of the department / unit	3 (34)
Fitness instructor	3 (34)
Cardiologist	1 (11)

### Community Readiness Scores

The overall readiness score was 3.28 (SD=0.30), which corresponded to the *vague awareness* stage of CR. The range of individual scores for each dimension of each interview was between 1.0 and 5.0. The highest average score of 3.72 (SD=0.79) was observed in the *Knowledge of Issue* dimension, whereas the *Knowledge of Efforts* domain received the lowest score of 2.92 (SD=1.59).

Table 3 represents consensus scores across each dimension based on all key informant interviews. The results suggested that four out of five dimensions were at *vague awareness* phase of CR. The only exception was the *Knowledge of Efforts* dimension, which was assessed to be at *denial/resistance* stage.

**Table 3.** Domain specific and overall community readiness scores

Dimension	Mean ± SD (Readiness Stage)
Community Knowledge of Issue	3.72 ± 0.79 (Vague Awareness)
Community Knowledge of Efforts	2.92 ± 1.59 (Denial/Resistance)
Community Climate	3.31 ± 1.22 (Vague Awareness)
Leadership	3.11 ± 0.86 (Vague Awareness)
Resources	3.33 ± 0.45 (Vague Awareness)
**Overall readiness**	**3.28 ± 0.30 (Vague Awareness)**

### Qualitative Assessment of Community Readiness

Dimension 1— Community Knowledge of Issue: This dimension addressed the scope of community members' knowledge and understanding of the issue of physical inactivity among senior citizens.

There was a common general understanding that physical activity was important in preserving physical and mental wellbeing, and hence was a significant factor in prolonging one's lifespan. However, when asked specifically about the issue of physical inactivity among seniors, informants (8 of 9) cited that there was lack of awareness. Healthy lifestyle and physical activity in particular were attracting the attention of younger generations but not among older adults.

“They know very little about it. Now there is a tendency that young people are interested in healthy nutrition and physical activity, but they do not inform their moms and dads, other adults.”

Head of Physical Therapy Unit, Hospital 2

According to three informants, older adults in the community may consider physical activity too risky or inappropriate for older age.

“As they have more health concerns, they become less active. They believe that physical movement can damage their joints, allocated time for each exercise might not be enough, and as if from the side they will look funny doing those activities.”

Deputy Director, Social Services Center

One interviewee believed this view was shared by younger generations as well when they thought that engagement in physical activities might exacerbate their parents’ and grandparents’ current conditions and lead to complications. A number of participants (3 of 9) including representatives from a hospital and fitness facilities pointed out that physical activity was often seen by older adults only as a supplement to medications and conventional medicine. Seniors may have underestimated the importance of exercise, for example, compared to following a medication regime. Two informants noted that this misconception was also present among healthcare professionals. Main discussion during a patient visit was around clinical concepts of disease, its symptoms, and conventional treatment methods. A respondent went on to explain:

“Doctors are not particularly familiar with sports either. To someone who has been living a sedentary life for 40 years they say, ‘You need to be physically active,’ and expect him/her to go straight into running.”

Instructor of Nordic Walking, Independent Provider

Dimension 2— Community Knowledge of Efforts: This dimension was comprised of how much community members knew about local efforts, if any, their effectiveness, and accessibility for older adults.

Efforts from the government, private, and nonprofit sectors aimed at increasing older adult physical activity were fragmented and rarely present. Three out of nine informants could not name any physical activity programs offered specifically for seniors. Nordic walking was the only example of a structured physical activity program mentioned by several interviewees (5 of 9).

“I have not seen a separate program specifically designed for the elderly - not from the private, nor from the public sector. A lot of different activities are carried out all around the city during summer months, but nothing is specific to older adults.”

Instructor of Physical Exercise Class, Fitness Center

Some fitness centers provided discounts for senior citizens to encourage the use of sports and leisure facilities. However, informants (3 of 9) believed that older adults often thought that fitness centers were for younger people, and it might be challenging for the elderly.

Those who were involved in designing or delivering services or heard about physical activity opportunities for senior citizens were uncertain of how widespread those activities were and if they were popular among older adults. According to informants (4 of 9), majority of community members were unaware or had limited knowledge about current and future efforts.

“We can see this by the way people call and come to us. They say, ‘We have elderly parents, and we do not know what to do with them.’ They ask for some kind of activities and events, but we are not aware of anything like that.”

Director, Older Adult Organization

Dimension 3— Community Climate: This dimension included the prevailing attitude of community members towards older age and the role of physical activity in older adulthood.

Informants indicated that older adult life was traditionally considered as a period to be spent with family, grandchildren, and relatives where older adult physical activity was of little value to seniors themselves and their children. After the age of 60, people may have become more heavily centered on family and household needs. Hence, their physical activity has been limited to everyday chores, such as grocery shopping and housekeeping.

According to informants, older adults were respected in the family and community. Their rare participation in physical activities was usually welcomed, but they were not seen as active members of society. As cited by one key informant, TV commercials portrayed older people as barely moving individuals. Another respondent suggested to think about common gifts that seniors received on birthdays and other occasions, and explained it in the following way:

“In our society, people very often present handkerchiefs, slippers, pajamas to older adults - all these gifts indicate that our view of the elderly is of an isolated and closed person. So even with our gifts we push them in the corner. If the community members saw them as an active part of society, then they would be given sports shoes, a sports bag, ice skating gear, active outfit.”

Director, Older Adult Organization

Informants acknowledged community concern about the issue of physical inactivity among older adults was limited to a group of enthusiasts and certain professionals, whereas the community as a whole expressed no concern and considered it to be an individual level problem. Informants (5 of 9) were skeptical about the problem becoming a real concern for city residents in near future.

“Our society is not ready to act on it yet. Now fitness is experiencing some kind of a boom and maybe involvement of older adults will somehow be a priority in the future, but it is not happening now. Probably, not going to happen soon.”

Instructor of Nordic Walking, Independent Provider

Dimension 4— Leadership: This dimension aimed to understand the position of the appointed leaders and influential community members in relation the issue and assessed their willingness to support current as well as future community actions.

Informants indicated that while the issue may have been a concern for the leadership, they showed no immediate motivation to act. Due to many other pressing issues needing attention and funding, older adult physical activity was not a priority. A respondent pointed out that the government's view of senior population was limited to pension reforms.

“When you ask about what is being done in relation to senior citizens, they [government officials] start explaining pension reforms. An elderly person is equal to pension affairs, and no one notices other aspects of older adult life.”

Director, Older Adult Organization

Dimension 5— Resources: The last dimension of the CRT explored the availability of local resources – human, money, and space – for community members to use in support of efforts now and in the future.

Informants (6 of 9) cited lack of financial resources as one of the most common barriers for seniors to engage in physical activities. Free opportunities had been accessible on a limited or seasonal basis, whereas fitness center memberships were unaffordable for the elderly for whom pension was usually the single source of income. Government funding was very scarce, and most of older adult physical activities were self-funded or supported by family members. When asked about alternative means of funding from grants and businesses, key informants (7 of 9) were unable to provide examples and explained that very limited financial support was available for promotion of older adult physical activity.

While not readily available, informants suggested that volunteers could be found among community members. To them, youth were especially involved in volunteering, which might help in activating older adults. In order to direct volunteer efforts towards effective promotion of physical activity among seniors, informants indicated that someone needed to train, organize, and mobilize them in a structured way.

“It seems to me that the problem lies precisely in the absence of an organizer or a leader who will be able to take the ownership of the problem, advocate for a solution, and use his/her skills as well as networks for the benefit of seniors… Volunteers can be easily recruited if there is a leader.”

Head of Physical Therapy Unit, Hospital 2

In terms of expert help, there appeared to be a sufficient number of knowledgeable and skilled healthcare workers, public health practitioners, and fitness instructors in the community. Yet, they may not all be trained to work specifically with older adult population. Two key informants mentioned that special training, expertise, and desire were needed to successfully accomplish such work.

Informants explained that during summer months, outdoor spaces could easily be used for physical activity sessions, but indoor spaces may not be readily available free of charge. Availability would also depend on the size of a required space. Throughout colder periods of the year, lack of indoor facilities in close proximity was thought to be a substantial barrier to physical activity.

## Discussion

We anticipated that the local community might score low in its readiness to challenge the current situation in relation to physical inactivity among senior citizens. In fact, quantitative assessment suggests that CR in Nur-Sultan is equal to the score of 3.28 on a nine-point scale, which is equivalent to the stage of *vague awareness*. According to the CRT, this means that most people recognize the issue as a local concern, but there is no immediate motivation to act on it. Qualitative analysis, however, indicates that community awareness might even be more limited and only certain groups such as fitness instructors and gerontologists express genuine concern regarding the issue. Such qualitative research findings suggest a *denial/resistance* stage of CR and highlight the importance of conducting in-depth analysis of interviewee responses in addition to calculating readiness scores when using the CRT. This also suggests that a broader assessment of CR with a greater number of key informants is needed in the future.

The literature points to many factors affecting engagement of older adults in physical activity.^21^,^22^. While identification of barriers was not a specific goal of the study, through qualitative analysis we identified a number of obstacles that prevent seniors from leading active lifestyles. Physical activity is often perceived by community members, including the elderly, as a pursuit of younger people, which could be risky or inappropriate for older adults. There is also an emphasis on conventional treatment of health problems and a disease-centric view of aging. Such beliefs, coupled with lack of information about the issue, appear to contribute to high levels of physical inactivity among seniors in Nur-Sultan.

Another common barrier is unaffordability of physical activity classes in the capital city. As evidenced by the results of the World Values Survey,^20^ only around 16% of older adults in Kazakhstan are able to save money and the rest “just get by” or “borrow money”. This fact may justify a low priority of physical activity among seniors. Likewise, the country and city leadership do not recognize the problem as a priority and have been passive in offering a solution. Lack of concern about the issue in the community and among the leadership could explain the scarcity of efforts. However, it is important to examine the current situation while taking into account the broader country profile. As a developing nation, Kazakhstan's government seems to be focusing on improving and diversifying the economy, and people are still driven by survival values of ensuring financial and physical security.^20^ In addition, the proportion of seniors in the country (7.4%) is significantly lower than in Europe (20%) and the US (16%),^23^ which could be distancing attention from the issues of older age, including physical inactivity. At the same time, high mortality and morbidity rates from cardiovascular diseases may in the near future incentivize the government to invest more resources in preventative initiatives.

The authors of the CRT suggest directing the efforts at increasing awareness, empowering community groups, and acquiring local support in order to move the readiness beyond the stage of vague awareness. For that purpose, studies from the US and China^12^,^14^ have recommended disseminating information about the benefits of physical activity through various printed and electronic media. In this regard, the government of Kazakhstan can use its vast network of state owned or regulated media channels to inform the community about the issue and to promote greater involvement of seniors in physical activity.

In the key informant interviews, Nordic walking was a commonly mentioned physical activity program. While it is not targeted at older adults, the program has been proven to be effective in improving heart rate, oxygen consumption, and quality of life.^24^ Promoting this program further may be a valid strategy to increase physical activity among older adults. Alternatively, evidence-based older adult activity programs such as Enhance®Fitness may be introduced in the community.^25^ However, implementation in Kazakhstan would first require a study of feasibility and necessary adaptations.

Given that the country's governance structure is highly centralized,^26^ national policies are likely to be more effective in changing the current situation. For instance, inclusion of older adult physical activity promotion as one of the priorities in the next State Healthcare Development Program^27^ may facilitate the promotion of older adult physical activity and the adoption of evidence-based programs throughout the country. This in turn could aid the transition from a disease-centric clinical paradigm towards a more prevention-centric, whole-person view of health care.

To our knowledge, this was the first effort to conduct a systematic evaluation of CR in Central Asia. Therefore, the concepts and methods of the study could be used by local researchers to conduct readiness assessments for other health and social problems in the communities of the region. Furthermore, in the vast majority of studies the CRT was used exclusively in a quantitative manner, whereas we went beyond that approach and included a qualitative assessment of key informant responses, which is a major strength of the study. Our final sample, however, included only 9 respondents. Although we ensured inclusion of representatives from a diverse range of fields, the study would have benefited from a larger sample size and a greater number of participants aged over 60. In addition, the concept of CR is community-specific, which limits the generalizability of the findings.
